# Supplementary figures and images for: Exonuclease ISG20 inhibits human cytomegalovirus replication by inducing an innate immune defense signature
Source: PLoS Pathog. 2026 Jan 9;22(1):e1013856. doi: 10.1371/journal.ppat.1013856 (PMC12818739; doi:10.1371/journal.ppat.1013856)

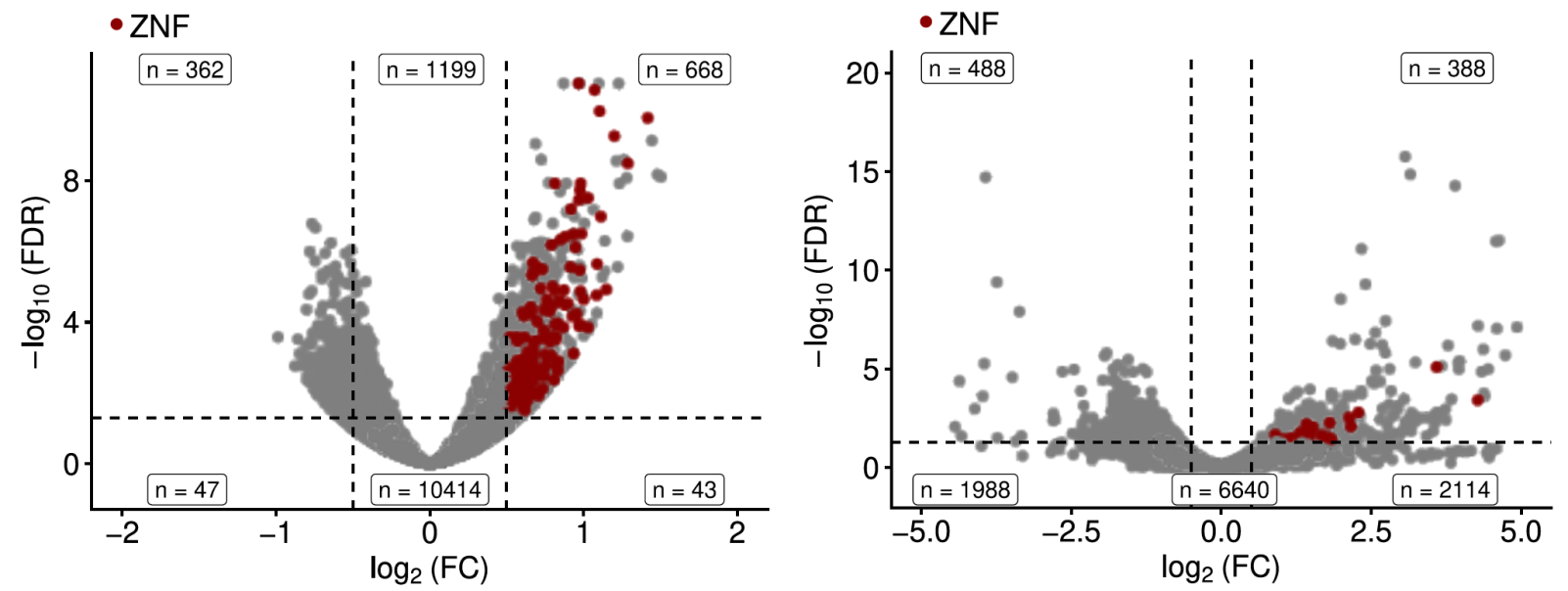

Supplement: S5 Fig — Volcano plots showing differentially expressed genes in HEK293T (A) and MEF (B) cells. Colored dots represent significantly regulated ZNF genes with adjusted p-value <0.05 and |log2 fold change| >0.5. (TIF) [file ppat.1013856.s005.tif]
