# Supplementary material for: Exonuclease ISG20 inhibits human cytomegalovirus replication by inducing an innate immune defense signature
Source: PLoS Pathog. 2026 Jan 9;22(1):e1013856. doi: 10.1371/journal.ppat.1013856 (PMC12818739; doi:10.1371/journal.ppat.1013856)
Supplement: S1 Table — (DOCX) [file ppat.1013856.s006.docx]

## **S1 Table. Key resources table**

| Key Resources Table | | | | |
| --- | --- | --- | --- | --- |
| Reagent type (species) or resource | Designation | Source or reference | Identifiers | Additional information |
| strain, strain background (Human cytomegalovirus) | AD169 | [1] |  | based on BAC HB15 |
| strain, strain background (Human cytomegalovirus) | TB40/E | [2] |  | based on TB40E-Bac4 |
| genetic reagent (Human cytomegalovirus) | AD169ΔIE1 | [3] |  | based on BAC HB15 |
| genetic reagent (Human cytomegalovirus) | TB40/E-UL84-luc | this paper |  | based on TB40E-Bac4 |
| genetic reagent (Herpes Simplex Virus Type I) | HSV-1-GFP | [4] |  | expresses a GFP-VP22 fusion protein |
| Cell line (Homo sapiens, human kidney) | HEK293T | DSMZ-German Collection of Microorganisms and Cell Cultures Gmbh | Cat# ACC 635 |  |
| antibody | mAb-beta-Actin (mouse monoclonal) | Sigma-Aldrich | Cat#A5441, RRID:AB_476744 | WB (1:10000) |
| antibody | mAb-IE1 p63-27 (mouse monoclonal) | [5] |  | WB (1:100) |
| antibody | mAb-MCP 28-4 (mouse monoclonal) | [6] |  | WB (1:2) |
| antibody | mAb-pp28 41-18 (mouse monoclonal) | [7] |  | WB (undiluted) |
| antibody | mAb-pp71 2H10-9 (mouse monoclonal) | [8] |  | WB (1:100) |
| antibody | mAb-TRIM28 (mouse monoclonal) | Abcam | Cat#ab22553,  RRID:AB_447151 | WB (1:500) |
| antibody | mAb-UL44 BS 510 (mouse monoclonal) | [9] |  | WB (1:5000) |
| antibody | mAb-UL69 69-66 (mouse monoclonal) | [10] |  | WB (1:10) |
| antibody | pAb-IE2 pHM178 (rabbit polyclonal) | [11] |  | WB (1:5000) |
| antibody | pAB-ISG20 | Thermo Fisher Scientific | Cat#PA5-30073,  RRID:AB_2547547 | WB (1:1000) |
| antibody | pAb-PML A301-167A (rabbit polyclonal) | Bethyl Laboratories | Cat#A301-167A,  RRID: AB_873108 | WB (1:5000) |
| antibody | pAb-PML A301-168A (rabbit polyclonal) | Bethyl Laboratories | Cat#A301-168A,  RRID: AB_2284081 | WB (1:5000) |
| antibody | pAb-SAMD9 (rabbit polyclonal) | Sigma-Aldrich | Cat#HPA021319, RRID:AB_1856554 | WB (1:1000) |
| antibody | pAb-ZAP (rabbit polyclonal | Thermo Fisher Scientific | Cat#PA5-31650,  RRID:AB_2549123 | WB (1:5000) |
| recombinant DNA reagent (plasmid) | pInducer20-CRSmut | [12] |  | lentiviral vector for inducible expression, mutated cis-repression signal |
| recombinant DNA reagent (plasmid) | pInducer20-CRSmut-ISG20 | this paper |  | inducible ISG20 expression |
| recombinant DNA reagent (plasmid) | pInducer20-CRSmut-ISG20mut | this paper |  | inducible ISG20mut expression |
| recombinant DNA reagent (plasmid) | pLenti-EF1a-C-mGFP-P2A-Puro | Origene | Cat#PS100121 | lentiviral vector for stable expression of mGFP-fused proteins |
| recombinant DNA reagent (plasmid) | pLenti-EF1a-ISG20-mGFP | this paper |  | stable expression of ISG20 |
| recombinant DNA reagent (plasmid) | pLenti-EF1a-ISG20mut-mGFP | this paper |  | stable expression of ISG20mut |
| chemical compound, drug | Actinomycin D | Sigma-Aldrich | Cat#A1410 |  |
| chemical compound, drug | Doxycycline-Hyclate | Sigma-Aldrich | Cat#D5207 |  |
| chemical compound, drug | Foscarnet | Sigma-Aldrich | Cat#PHR1436-1G |  |
| chemical compound, drug | Interferon-alpha | R&D Systems | Cat#11101-1 |  |
| chemical compound,  drug | Interferon-beta | R&D Systems | Cat#11415-1 |  |
| chemical compound,  drug | Interferon-gamma | Sigma-Aldrich | Cat#285-IF |  |
| chemical compound,  drug | Ruxolitinib | AdipoGen Life Sciences | Cat#AG-CR1-3624-M005 |  |

**References:**

1. Hobom U, Brune W, Messerle M, Hahn G, Koszinowski UH. Fast screening procedures for random transposon libraries of cloned herpesvirus genomes: mutational analysis of human cytomegalovirus envelope glycoprotein genes. J Virol. 2000;74(17):7720-9.

2. Sinzger C, Hahn G, Digel M, Katona R, Sampaio KL, Messerle M, et al. Cloning and sequencing of a highly productive, endotheliotropic virus strain derived from human cytomegalovirus TB40/E. JGenVirol. 2008;89(Pt 2):359-68.

3. Scherer M, Otto V, Stump JD, Klingl S, Muller R, Reuter N, et al. Characterization of Recombinant Human Cytomegaloviruses Encoding IE1 Mutants L174P and 1-382 Reveals that Viral Targeting of PML Bodies Perturbs both Intrinsic and Innate Immune Responses. J Virol. 2016;90(3):1190-205. Epub 2015/11/13. doi: 10.1128/JVI.01973-15

JVI.01973-15 [pii]. PubMed PMID: 26559840; PubMed Central PMCID: PMC4719593.

4. Elliott G, O'Hare P. Live-cell analysis of a green fluorescent protein-tagged herpes simplex virus infection. JVirol. 1999;73(5):4110-9.

5. Andreoni M, Faircloth M, Vugler L, Britt WJ. A rapid microneutralization assay for the measurement of neutralizing antibody reactive with human cytomegalovirus. J VirolMethods. 1989;23(2):157-67.

6. Waldo FB, Britt WJ, Tomana M, Julian BA, Mestecky J. Non-specific mesangial staining with antibodies against cytomegalovirus in immunoglobulin-A nephropathy. Lancet. 1989;1(8630):129-31.

7. Sanchez V, Sztul E, Britt WJ. Human cytomegalovirus pp28 (UL99) localizes to a cytoplasmic compartment which overlaps the endoplasmic reticulum-golgi-intermediate compartment. JVirol. 2000;74(8):3842-51.

8. Kalejta RF, Bechtel JT, Shenk T. Human cytomegalovirus pp71 stimulates cell cycle progression by inducing the proteasome-dependent degradation of the retinoblastoma family of tumor suppressors. MolCell Biol. 2003;23(6):1885-95.

9. Plachter B, Nordin M, Wirgart BZ, Mach M, Stein H, Grillner L, et al. The DNA-binding protein P52 of human cytomegalovirus reacts with monoclonal antibody CCH2 and associates with the nuclear membrane at late times after infection. Virus Res. 1992;24(3):265-76.

10. Winkler M, Rice SA, Stamminger T. UL69 of human cytomegalovirus, an open reading frame with homology to ICP27 of herpes simplex virus, encodes a transactivator of gene expression. JVirol. 1994;68(6):3943-54.

11. Hofmann H, Floss S, Stamminger T. Covalent modification of the transactivator protein IE2-p86 of human cytomegalovirus by conjugation to the ubiquitin-homologous proteins SUMO-1 and hSMT3b. JVirol. 2000;74(6):2510-24.

12. Schilling EM, Scherer M, Rothemund F, Stamminger T. Functional regulation of the structure-specific endonuclease FEN1 by the human cytomegalovirus protein IE1 suggests a role for the re-initiation of stalled viral replication forks. PLoS Pathog. 2021;17(3):e1009460. Epub 2021/03/27. doi: 10.1371/journal.ppat.1009460. PubMed PMID: 33770148; PubMed Central PMCID: PMCPMC8026080.
