# Supplementary material for: Exonuclease ISG20 inhibits human cytomegalovirus replication by inducing an innate immune defense signature
Source: PLoS Pathog. 2026 Jan 9;22(1):e1013856. doi: 10.1371/journal.ppat.1013856 (PMC12818739; doi:10.1371/journal.ppat.1013856)
Supplement: S2 Table — (DOCX) [file ppat.1013856.s007.docx]

**S2 Table: Oligonucleotides**

| Oligonucleotides for cloning and sequencing of ISG20 in pInducer20-CRSmut | |
| --- | --- |
| 5’ISG20_attB1 | 5’-GGG GAC AAG TTT GTA CAA AAA AGC AGG CTA TGG CTG GGA GCC GTG AG-3’ |
| 3’ISG20_attB2 | 5’-GGG GAC CAC TTT GTA CAA GAA AGC TGG GTC TTA GTC TGA CAC AGC CAG GCG-5’ |
| 5’-pInducerseq | 5’- CCA TCC ACG CTG TTT TGA CC-3’ |
| Oligonucleotides for cloning and sequencing of ISG20 in pLenti-EF1a-C-mGFP-P2A-Puro | |
| 5’ISG20_SgsI | 5’- CAT AGG CGC GCC ATG GCT GGG AGC CGT GAG -3’ |
| 3’ISG20_MluI | 5’- CAT AAC GCG TGT CTG ACA CAG CCA GGC G -3’ |
| EF51-F | #GE100088 (Origene) |
| Oligonucleotides for real-time PCR (TaqMan) | |
| 5’CMV | 5’- AAG CGG CCT CTG ATA ACC AAG -3’ |
| 3’CMV | 5’- GAG CAG ACT CTC AGA GGA TCG G -3’ |
| CMV-FAM/TAMRA | 5’-CAT GCA GAT CTC CTC AAT GCG GCG-3’ |
| 5’Alb | 5’-GTG AAC AGG CGA CCA TGC T-3’ |
| 3’Alb | 5’- GCA TGG AAG GTG AAT GTT TCA G -3’ |
| Alb-FAM/TAMRA | 5’- TCA GCT CTG GAA GTC GAT GAA ACA TAC GTT C -3’ |
| Oligonucleotides for real-time PCR (SYBR Green), viral genes | |
| 5’HCMV IE2 | 5’- TGA CCG AGG ATT GCA ACG A -3’ |
| 3’HCMV IE2 | 5’- CGG CAT GAT TGA CAG CCT G -3’ |
| 5’HCMV UL35 | 5’- TTG CAG CTA CTG ACG CAA CT -3’ |
| 3’HCMV UL35 | 5’- TTC TCC TGC TCT TCG TCC TC -3’ |
| 5’HCMV UL44 | 5’- TTT TCT CAC CGA GGA ACC TTT C -3’ |
| 3’HCMV UL44 | 5’- CCG CTG TTC CCG ACG TAA T -3’ |
| 5’HCMV UL75 | 5’- GCA AAA GGC GCA GTT TTC TA -3’ |
| 3’HCMV UL75 | 5’- TCC TAC CCT GTC TCC ACC AC -3’ |
| 5’HCMV UL76 | 5’- AAG CAC CTG GAC ATC TAC CG -3’ |
| 3’HCMV UL76 | 5’- TCC GCC GAC TTA ATC GTA CT -3’ |
| 5’HCMV UL82 | 5’- ACG ACA CCG TAG ACC TGA CC -3’ |
| 3’HCMV UL82 | 5’- AAA GAG GTG CAG TCC GCT AA -3’ |
| 5’HCMV UL99 | 5’- GAG GAC AAG GCT CCG AAA C -3’ |
| 3’HCMV UL99 | 5’- CTT TGC TGA TGG TGG TGA TG -3’ |
| 5’HCMV US3 | 5’- CTG GAT GTG GTG GTA TCG GA -3’ |
| 3’HCMV US3 | 5’ TGT TTC TCG GTG AAG TTG CC -3’ |
| Oligonucleotides for real-time PCR (SYBR Green), cellular genes | |
| 5’IFIT2/ISG54 | 5’- ATG TGC AAC CTA CTG GCC TAT -3’ |
| 3’IFIT2/ISG54 | 5’- TGAGAGTCGGCCCATGTGATA -3’ |
| CCL-8 | Cat# VHPS-1625, Real-time primer set (Biomol) |
| GAPDH | Cat# VHPS-3541, Real-time primer set (Biomol) |
| IFIT1 | Cat# VHPS-4465, Real-time primer set (Biomol) |
| IFIT3/4 | Cat# VHPS-4466, Real-time primer set (Biomol) |
| IFN-ß1 | Cat# VHPS-4476 ,Real-time primer set (Biomol) |
| IRF3 | Cat# VHPS-4629, Real-time primer set (Biomol) |
| ISG20 | Cat# HP205936, Real-time primer set (Origene) |
| OASL | Cat# VHPS-6424, Real-time primer set (Biomol) |
| TNFSF10 | Cat# VHPS-9439, Real-time primer set (Biomol) |
