# Supplementary material for: Exonuclease ISG20 inhibits human cytomegalovirus replication by inducing an innate immune defense signature
Source: PLoS Pathog. 2026 Jan 9;22(1):e1013856. doi: 10.1371/journal.ppat.1013856 (PMC12818739; doi:10.1371/journal.ppat.1013856)
Supplement: S3 Table — (DOCX) [file ppat.1013856.s008.docx]

**S3 Table: ON-TARGETplus siRNA Smart Pools (Horizon Discovery)**

| **Gene** | **Gene ID** | **Gene Accession** | **Cat#** |
| --- | --- | --- | --- |
| *APOL2* | 23780 | NM_030882 | L-017407-00 |
| *APOL6* | 80830 | NM_030641 | L-013432-01 |
| *DDX58* | 23586 | NM_014314 | L-012511-00 |
| *DDX60* | 55601 | NM_017631 | L-017664-01 |
| *FGF2* | 2247 | NM_002006 | L-006695-00 |
| *HERC5* | 51191 | NM_016323 | L-005174-00 |
| *HERC6* | 55008 | NM_001013000 | L-005175-00 |
| *HIP1R* | 9026 | NM_003959 | L-027079-00 |
| *IFI44* | 10561 | NM_006417 | L-016368-00 |
| *IFIH1* | 64135 | NM_022168 | L-013041-00 |
| *IFIT1* | 3434 | NM_001001887 | L-019616-00 |
| *IFIT2* | 3433 | NM_001547 | L-012582-02 |
| *IFIT3* | 3437 | NM_001549 | L-017691-00 |
| *ISG15* | 9636 | NM_005101 | L-004235-03 |
| *ISG20* | 3669 | NM_002201 | L-015994-00 |
| *MX1* | 4599 | NM_002462 | L-011735-00 |
| *MX2* | 4600 | NM_002463 | L-011736-00 |
| *OAS2* | 4939 | NM_001032731 | L-009768-00 |
| *OASL* | 8638 | NM_198213 | L-012617-00 |
| *PARP14* | 54625 | NM_017554 | L-023583-00 |
| *PMAIP1* | 5366 | NM_021127 | L-005275-00 |
| *RARRES3* | 5920 | NM_004585 | L-011889-00 |
| *RNF149* | 284996 | NM_173647 | L-007169-00 |
| *RSAD2* | 91543 | NM_080657 | L-015423-00 |
| *SAMD9* | 54809 | NM_017654 | L-031141-00 |
| *SAMD9L* | 219285 | NM_152703 | L-028907-00 |
| *WARS* | 7453 | NM_213645 | L-008322-00 |
| *ZC3HAV1* | 56829 | NM_024625 | L-017449-01 |
| *ZFP36L2* | 678 | NM_006887 | L-013605-01 |
| *ZNFX1* | 57169 | NM_021035 | L-014074-00 |
| Non-targeting control pool | | | D-001810-10 |
